# Application of Nanofluids in Improving the Performance of Double-Pipe Heat Exchangers—A Critical Review

**DOI:** 10.3390/ma15196879

**Published:** 2022-10-03

**Authors:** Stephan Pierre Louis, Svetlana Ushak, Yanio Milian, Magdalena Nemś, Artur Nemś

**Affiliations:** 1 Center for Advanced Research in Lithium and Industrial Minerals (CELiMIN), Departamento de Ingeniería Química y Procesos de Minerales, Universidad de Antofagasta, Antofagasta 02800, Chile; 2 Department of Thermodynamics and Renewable Energy Sources, Faculty of Mechanical and Power Engineering, Wrocław University of Science and Technology, Wybrzeże Wyspiańskiego 27, 50-370 Wrocław, Poland

**Keywords:** nanofluids, nanoparticles, double-pipe heat exchanger, pressure drop, heat transfer coefficient, morphology

## Abstract

Nanofluids can be employed as one of the two fluids needed to improve heat exchanger performance due to their improved thermal and rheological properties. In this review, the impact of nanoparticles on nanofluid properties is discussed by analyzing factors such as the concentration, size, and shape of nanoparticles. Nanofluid thermophysical properties and flow rate directly influence the heat transfer coefficient and pressure drop. High thermal conductivity nanoparticles improve the heat transfer coefficient; in particular, metallic oxide (such as MgO, TiO_2_, and ZnO) nanoparticles show greater enhancement of this property by up to 30% compared to the base fluid. Nanoparticle size and shape are other factors to consider as well, e.g., a significant difference in thermal conductivity enhancement from 6.41% to 9.73% could be achieved by decreasing the Al_2_O_3_ nanoparticle size from 90 to 10 nm, affecting nanofluid viscosity and density. In addition, equations to determine the heat transfer rate and the pressure drop in a double-pipe heat exchanger are presented. It was established that the main factor that directly influences the heat transfer coefficient is the nanofluid thermal conductivity, and nanofluid viscosity affects the pressure drop.

## 1. Introduction

The double-tube heat exchanger is one of the most common designs of heat exchangers used in commercial and industrial applications. It is the simplest and one in which hot and cold fluids move in same or opposite directions [1]. A great advantage of the double-tube heat exchanger is the ability to process products with particles without any blockage risk.

The chemical, food, oil, and gas industries use double-pipe heat exchangers to perform tasks such as pasteurization, sterilization, reheating, preheating, digester heating, and effluent heating processes [2], for example, heating and/or cooling in sanitary and pharmaceutical applications. Moreover, the double-tube heat exchanger has been widely used in different renewable energy systems, such as solar energy, waste heat recovery, geothermal, combustion, latent heat energy storage, and air conditioning, due to its simple construction, easy cleaning, and low cost [3].

The heat transfer improvement in exchangers is usually accompanied by a pressure drop increase and, as a consequence, it requires higher pumping power. Therefore, any gains from improved heat transfer should be balanced against the associated pressure drop cost [4,5]. In this way, the main reasons for research work on heat exchangers are (i) to enhance their heat transfer rate, consequently reducing the heat exchanger’s overall size, saving initial cost and space, and (ii) to minimize or avoid a large pressure drop, allowing pumping power to be reduced and saving operating costs. Therefore, knowledge about the pressure drop and convective heat transfer characteristics in heat exchangers is essential for adopting this technology into marketable products. Therefore, several techniques are scrutinized in order to enhance heat pipe performance, such as new structure configurations, designs, and technologies or their modification, the integration of heat pipes with other systems such as solar concentrators, improving the working fluid type, or employing heat storage systems [6].

In the recent decade, many scientists have worked to improve performance in heat exchangers using nanofluids as heat transfer fluids. Kavitha et al. experimentally used the CuO-water nanofluid as a heat transfer fluid to enhance a double-pipe heat exchanger [7]. Jassim et al. experimentally assessed TiO_2_ and Al_2_O_3_ nanofluids on heat exchanger performance by considering nanofluids replacing conventional fluid, resulting in performance improvements from 13% to 23% with a concentration of 3% [8]. Ding et al. carried out a numerical simulation of TiO_2_-water nanofluids in a double-pipe heat exchanger considering various flow rates and TiO_2_ mass fractions. The results demonstrated that the heat transfer capacities of all mass fractions of TiO_2_-water nanofluids were higher than those of deionized water, but they also increased the flow resistance in a corrugated pipe [9]. Akbar et al. achieved excellent results employing a hybrid nanofluid (Al_2_O_3_+TiO_2_-water) to improve the heat transfer and pressure drop through horizontal tubes with diameter sizes of 30 to 45 nm. The results proved that the thermophysical properties of the hybrid nanofluid were enhanced from 7 to 13% compared to water, increasing the Nusselt number by approximately 30%, with a slight (5%) increase in pressure drop along a horizontal heated tube and a heat transfer highly appropriate for practical and industrial applications [10]. Jassim et al. experimentally assessed Al_2_O_3_ and Cu nanofluids on performance and heat leak in a double-pipe heat exchanger, showing that the Nusselt number was enhanced at all volume concentrations of Cu and Al_2_O_3_ nanofluids when compared to the base fluid (water). The Nusselt number was directly proportional to the Reynolds number for all cases. The use of nanoparticles in the base fluid causes an increment in exchanger effectiveness [11]. Mansoury et al. experimentally studied the heat transfer and flow characteristics of an Al_2_O_3_-water nanofluid in various heat exchangers on counter flow with a 20 nm nanoparticle size and turbulent flow. The double-pipe heat exchanger presented a 60% enhancement in the heat transfer coefficient, while the plate heat exchanger reflected an 11% increment in the heat transfer coefficient. However, the smallest percentage of pressure drop of 27% was reported in the plate heat exchanger, compared to the double-pipe heat exchanger at 85% [12].

Likewise, several studies confirmed simultaneous improvements in heat exchangers and solar collectors using a nanofluid as one of their heat transfer fluids. Vincely et al. performed an experimental investigation of solar flat plate collector performance using a graphene oxide–water nanofluid under forced circulation connected to a concentric-tube heat exchanger. It was observed that the collector efficiency was enhanced with increasing concentrations and flow rates. The heat transfer coefficient increments for the nanofluid in a laminar flow with concentrations of 0.005, 0.01, and 0.02 were 8.03%, 10.93%, and 11.50%, respectively [13]. Similarly, Henein et al. utilized a MgO/MWCNT-water hybrid nanofluid as a working fluid to enhance the thermal performance of a heat-pipe evacuated-tube solar collector with a 0.02% concentration at various volume flow rates ranging from 1 to 3 L/min. The results showed an enhancement in the energy and exergy efficiencies with an increase in the weight ratios of the MWCNT nanoparticles and the volume flow rate. The energy and exergy efficiency enhancements for the collector were 55.83% and 77.14%, respectively, for the MgO/MWCNT (50:50) hybrid nanofluid [14].

As already discussed, almost all research activities have focused solely on improving the heat transfer coefficient. However, they have not emphasized the importance of minimizing or avoiding pressure drops. Moreover, studies related to the use of nanofluids in heat exchangers only support factors affecting heat transfer, while few or no discussions about the parameters that influence pressure drop are deeply scrutinized, to the best of our knowledge. Consequently, the main objective of this review is to analyze the factors influencing nanofluids in the performance of heat exchangers, not only to improve heat transfer but also to minimize or avoid large increments in pressure drop.

## 2. Heat Exchanger Performance

Different methodologies were investigated to improve heat exchanger performance. Examples of active and passive methods are summarized in Table 1. For active methods, external forces are required for heat transfer performance in double-tube heat exchangers. Generally, the main external forces used were mechanical forces, ultrasound, and magnetic fields [3]. As can be seen in Table 1, high-power and sophisticated devices were applied to improve the heat transfer rate by active methods. Contrastingly, passive methods did not require a powerful force. In that case, simple techniques were used to improve the heat transfer coefficient. Helical wires and porous media were two techniques considered with a greater percentage of pressure drop. However, this pressure drop could be compensated with a percentage of heat transfer improvement. Passive techniques were better when compared to active techniques because of their simplicity, low cost, and certain level of enhancement with a tolerable pressure drop [15]. The use of nanoparticles has been one of the most promising ways among the passive methods to improve heat exchanger performance.

**Figure 1:**
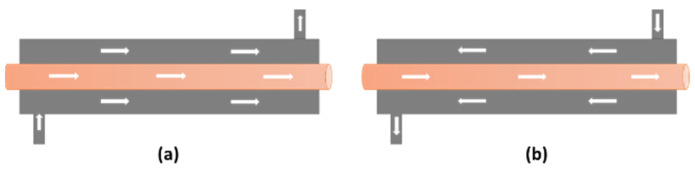
Double-pipe heat exchangers using (**a**) parallel flow and (**b**) counter flow [3].

Double-pipe heat exchanger performance consists of increasing the heat transfer rate and avoiding or minimizing a large pressure drop. Commonly, a fluid with a high viscosity value is the most appropriate for the side with the larger passage area (annular) since it implies a lower pressure drop [4]. In Figure 1, a diagram of a double-pipe heat exchanger is presented. When hot and cold fluids move in same or opposite directions in a double-pipe construction, this corresponds to the simplest heat exchanger model. One of the fluids passes through the smallest tube, while the other passes through the annular space between two tubes. Two types of flow arrangement are possible, one is employing both fluids in parallel flow, where cold and hot fluids enter at the same edges and move in the same direction (Figure 1a). In the other arrangement (Figure 1b), fluids enter at opposite inlets in counterflow, running in opposite directions [4,5].

**Figure 2:**
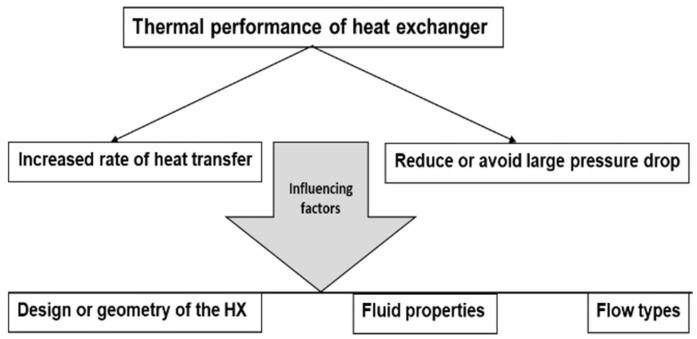
Scheme showing factors influencing heat exchanger performance.

The main factors that influence heat exchanger performance are the design, fluid properties, and flow types (Figure 2). A lower pressure drop helps avoid the requirement for a huge pump without affecting the initial cost. It also reduces electrical consumption, which affects operating costs. Low speeds are helpful to avoid erosion, tube vibrations, and noise as well as pressure drop [4].

A novel method that has been promoted recently is the use of nanofluids. Different nanofluids have been investigated to improve double-tube heat exchanger performance [26]. The heat transfer coefficient and the pressure drop are characterized by relative variations in nanofluid thermophysical properties that include the density, specific heat, viscosity, expansion coefficient, and thermal conductivity in addition to the flow regime [27]. According to Gupta et al., nanofluid thermophysical properties depend on the base fluid and on the influence of certain factors specific to nanoparticles, such as the concentration, size, and geometry (shape) [28]. In the next section, a detailed analysis of this topic is provided.

## 3. Impact of Morphology and Concentration of Nanoparticles on Nanofluid Properties

### 3.1. Nanofluids in Heat Exchangers

Nanoparticles (NPs) are particulate substances that range between 5 and 100 nm in size [29]. Recently, nanoparticles have been used in a base fluid for the formation of new materials called nanofluids for special uses in many applications such as solar energy [30,31], automotive radiators [32,33], thermal energy storage [34,35], refrigeration [36,37], and double-pipe heat exchangers (Table 2).

These nanofluids can be employed as heat transfer fluids that usually have a higher thermal performance than other conventional fluids. They have been investigated for a long time as an alternative working fluid. They are formed from the suspension of small solid particles of nanometer size in a base fluid. Conventional fluids that are usually employed as base fluids are water, ethylene glycol, engine oil, paraffin oil, and others [38].

**Table 2:** Formed nanofluids commonly used in the enhancement of double-pipe heat exchangers.

Nanoparticles	Size (nm)	Base Fluid	Concentration (%)	Temperature Range (°C)	Observation	Ref.
Al_2_O_3_	45	60% ethylene glycol 40% water	4.00	11.85–151.85	Average heat transfer enhancements of 40%, 29%, and 43%, respectively. Increased pressure drop by 84%, 47%, and 100%, respectively.	[39]
SiO_2_	50					
CuO	29					
TiO_2_	8–18	Water	0.25	30–60	Heat transfer coefficient enhanced by up to 32%, 21%, and 16%, respectively.	[40]
ZnO	12–28					
Ag	7–24					
Multiwalled carbon nanotubes (MWCNT)	20–30	Solar glycol	0–0.60	30–50	Heat transfer enhanced by 115% for 0.04 kg/s and 0.6% concentration. A 1.56-fold increase in pressure drop for 0.08 kg/s and 0.6% concentration.	[41]
Fe_3_O_4_	50–100	Water	0–0.40	31–90	Heat transfer enhanced by 80–90% for 0.4% concentration. Increase in pressure drop, along with the Reynolds number and the nanofluid volume concentration.	[21]
MgO	45–50	50% water—50% ethylene glycol	0–0.300	20–100	Heat transfer enhanced by 39% at wt.% = 0.3 and friction factor increased by 33.80%.	[42]
TiO_2_	21	Water	0.2	15–50	Heat transfer coefficient enhanced by 6–11%. Low penalty of pressure drop.	[43]

It can be observed that nanoparticle characteristics have a significant impact on nanofluid properties and influence the heat transfer coefficient. High thermal conductivity nanoparticles are better since this feature improves both the nanofluid and its heat transfer coefficient. Nanoparticles such as metallic oxides show greater heat transfer coefficient enhancement than others. TiO_2_ and ZnO nanoparticles result in better heat transfer percentage improvements than Ag nanoparticles. A large observed pressure drop value corresponds to a high concentration and a flow rate increment. In the next section, other than nanoparticle nature, additional parameters that influence nanofluids are detailed.

### 3.2. Influences of Nanoparticles on Nanofluid Properties

A nanoparticle suspension in a base fluid enhances the energy transmission in the fluid, leading to improved thermal conductivity properties and better heat transfer characteristics [26]. According to Pordanjani et al., there are four thermophysical properties of nanofluids that change with the addition of nanoparticles to a base fluid: the density, viscosity, thermal conductivity, and specific heat [44]. Moreover, other experimental studies have demonstrated that nanofluid thermophysical properties depend on the concentration, size, shape, and characteristics (nature) of nanoparticles [38]. Hozien et al. have experimentally studied the thermophysical properties of TiO_2_/water, ZnO/water, and Ag/water nanofluids, with their average nanoparticle sizes being 14, 20, and 16 nm, respectively. Three types of nanofluids were prepared with nanoparticle volume concentrations of 0.25%. Their results showed that the density, viscosity, and thermal conductivity of all three nanofluids increased. Precisely, the thermal conductivity values showed average enhancements of 8.50%, 6.00%, and 5.00%, respectively. XRD, SEM, TEM, and the Zetasizer test are used for the characterization of synthesized nanoparticles [40]. In the following sections, the influences of the most significant factors (volume concentration, size, and shape) on the thermophysical properties of nanofluids are presented.

#### 3.2.1. Influences of Volume Concentration of Nanoparticles on Nanofluids Properties

**Figure 3:**
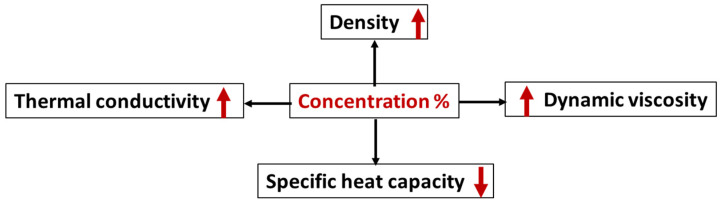
Influences of an increase in nanoparticle concentration in a base fluid on nanofluid properties.

In Figure 3, the effect of the nanoparticle concentration on nanofluid properties is presented. In general, an increasing particle concentration in a base fluid increases the nanofluid viscosity and density while enhancing its thermal conductivity. Experimental studies with a Viscolite 2700 viscometer carried out by Rabienataj et al. showed that a nanoparticle concentration increase in base a fluid increases the nanofluid viscosity [45]. Similarly, Osman et al. measured the nanofluid viscosity of (Al_2_O_3_—water), resulting in an increase in nanofluid viscosity with the volume concentration [46]. Later, with a new experimental study of nanofluids (Al_2_O_3_-base fluid and CuO-base fluid), Asokan et al. confirmed that the density, thermal conductivity, and viscosity increase with the nanoparticle concentration in the base fluid [47]. A mixture of 60% ethylene glycol and 40% water was used as a base fluid [48]. Recently, Saleh et al. analyzed a MWCNT/water nanofluid (multiwalled carbon nanotube/water nanofluids), demonstrating that the thermal conductivity, viscosity, and density were augmented by 15.27%, 9.15%, and 1%, respectively, at a 0.3% particle concentration compared with water as the base fluid. The characterization of MWCNT was performed with XRD (Siemens D-500, 45 kV) and SEM (Hitachi SU-70 SEM) [49]. The nanofluid density was the property that influenced the Reynolds number, determining the flow condition (laminar or turbulent), and allowed for the heat transfer coefficient and pressure drop calculations [50]. Then, the density’s influence on heat exchanger performance was not significant because it exerted a positive influence on the heat exchanger by enhancing the heat transfer coefficient and a negative one by increasing the pressure drop percentage.

In Table 3, a summary of examples of the effects of the nanoparticle volume fraction on nanofluid properties is presented. Generally, it could be observed through the articles reviewed in this section that nanofluids’ main thermophysical properties were influenced by the modification of nanoparticle concentrations in the base fluids. With a concentration increase, thermal conductivity, density, and viscosity increased. Metal oxide nanoparticles showed better behavior in thermal conductivity compared with metallic nanofluids. Metallic nanoparticles presented better improvements in density and viscosity. The nanofluid specific heat was not significant in the process because it resulted in lower performance than the base fluid. It should be remembered that, with increasing temperature, nanofluid thermal conductivity increases and viscosity and density decrease.

#### 3.2.2. Influences of Nanoparticle Size on Nanofluid Properties

**Figure 4:**
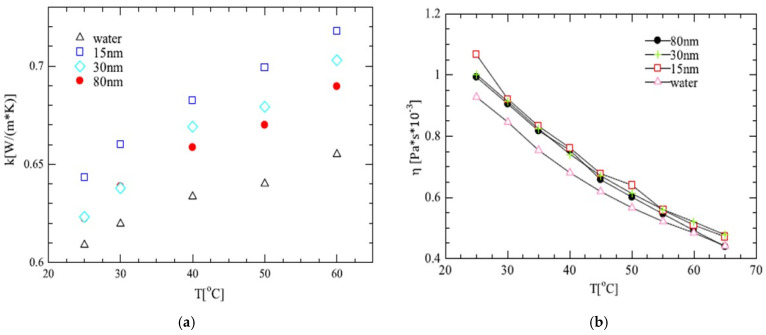
Variations in the thermal conductivities (**a**) and the dynamic viscosities (**b**) of the SiO_2_−water nanofluids as a function of temperature and particle size [54].

Nanoparticle size is an important parameter that influences the main thermophysical properties of nanofluids. Nanoparticles can be synthesized in various sizes ranging from 5 to 100 nm. Kim et al. experimentally studied the performance improvement of a U-tube solar collector, depending on the nanoparticle size and the concentration of an Al_2_O_3_-water nanofluid. A volume fraction of 1% and nanoparticles of three diameters (20, 50, and 100 nm) were used. It was observed that the thermal conductivity enhancement at a 20 nm nanoparticle size was approximately 1.54% and 2.43% higher than those at 50 nm and 100 nm nanoparticle sizes, respectively. At the same nanofluid concentration, the maximum efficiencies of the solar collector with 20, 50, and 100 nm nanoparticles in the nanofluid were 24.10%, 20.40%, and 17.80%, respectively, higher than that with water. The overall number of small nanoparticles was greater than that of large nanoparticles. An ultrasonic oscillation apparatus (SHT 750S, 750 W power, 19.97 kHz frequency) and SEM were used for the characterization and preparation of the nanofluids [53]. Then, Zhang et al. studied the effects of particle size on the heat transfer performance of SiO_2_-water nanofluids. Good suspension stability and dispersion were prepared, and their thermal conductivities were measured using the transient hot wire method. The results showed that the SiO_2_−water nanofluid thermal conductivities with particle sizes of 15, 30, and 80 nm were 7.80, 4.90, and 3.80% higher than those of water, respectively, which meant that the SiO_2_−water nanofluids thermal conductivity was higher than water. It was also observed that smaller nanofluid nanoparticle sizes obtained higher dynamic viscosity values than the base fluid. An FS-1200 ultrasonic processor and a Minizeta 03E laboratory horizontal grinder were the apparatus for nanofluid preparation (Figure 4) [54]. Similarly, Main et al. experimentally assessed the nanoparticle size effects on the density, viscosity, and thermal conductivity of ionic liquid (IL)-based nanofluids. In that work, 1 wt.% aluminum oxide (Al_2_O_3_) nanoparticles with different particle sizes (10 nm, 30 nm, 60 nm, and 90 nm) were mixed in base ionic liquids with a working temperature range of 10 °C to 70 °C. It was noticed that the average thermal conductivity enhancements were 9.73%, 6.53%, 6.41%, and 7.60% for 10 nm, 30 nm, 60 nm, and 90 nm nanoparticles, respectively. A Brookfield DV3T viscometer and a KD2 Pro thermal property analyzer were used for the measurement of nanofluid properties [55].

Nanoparticle size influences the viscosity, thermal conductivity, and density of nanofluids. Decreasing the nanoparticle size enhances nanofluid thermophysical properties [28,54]. Additionally, the main factors affecting nanofluid thermophysical properties include nanoparticle morphology and concentration [26].

In this section, based on a review of previous research, it is clearly observed that the smallest nanoparticles presented an improvement in thermal conductivity and viscosity and no significant variation in density (Table 4). Next, nanoparticle shape’s influence on nanofluid properties is analyzed.

#### 3.2.3. Influences of Nanoparticle Shape on Nanofluid Properties

**Figure 5:**
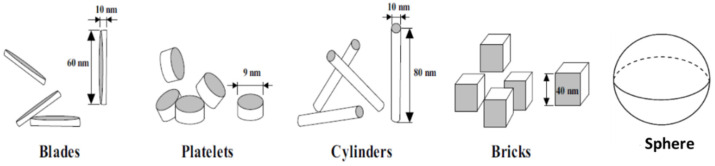
Nanoparticle shapes commonly used in base fluids to form nanofluids [57].

The nanoparticle shapes commonly used in base fluids (Figure 5) to form nanofluids were shown to directly affect only the viscosity and thermal conductivity of the final nanomaterials [28].

**Figure 6:**
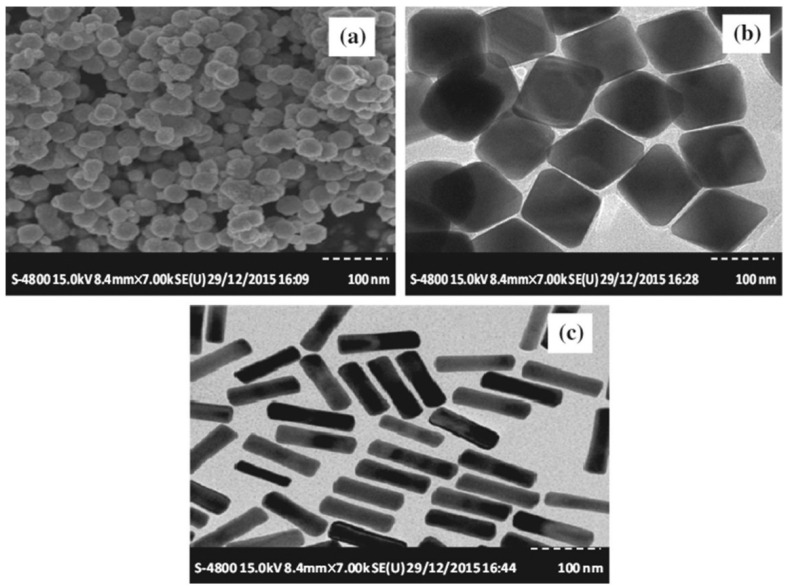
TiO_2_ nanoparticle SEM images of (**a**) spherical, (**b**) brick (cubic), and (**c**) cylindrical (rod) shapes [57].

Zahmatkesh et al. presented both formulas and different shape factors to determine the thermal conductivity and viscosity of nanofluids (see Figure 6) [57].

**Figure 7:**
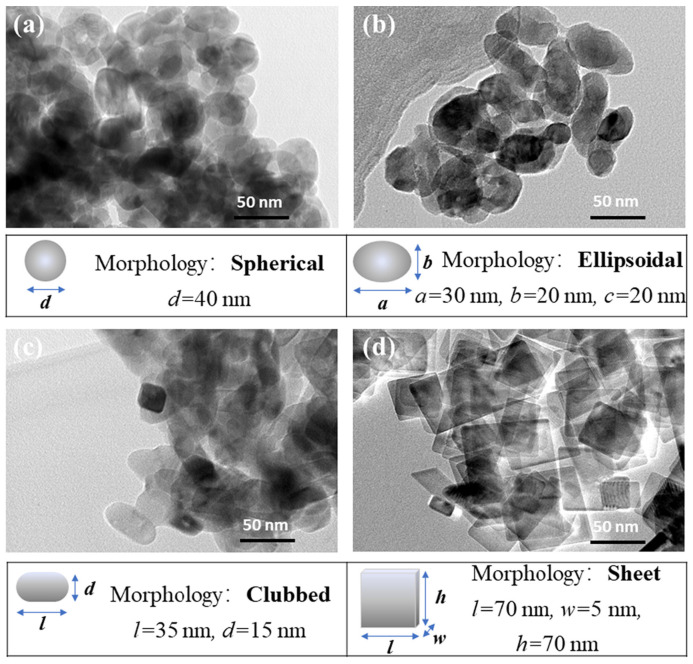
TEM images and morphology descriptions for TiO_2_ nanoparticles with different shapes: (**a**) spherical, (**b**) ellipsoidal, (**c**) clubbed, and (**d**) sheet [58].

Cui et al. experimentally carried out a study on the thermal conductivity of nanofluids with different nanoparticle shapes, as presented in Figure 7. The results showed that the relative thermal conductivity (RTC) of TiO_2_/water nanofluids with clubbed and sheet-shaped nanoparticles is higher than other shapes, and TiO_2_ nanofluids with sheet nanoparticles showed the highest RTC for a temperature of 60 °C and a nanoparticle concentration of 4%. A transmission electron microscope (TEM, JEM-1200EX, Jeol Ltd., Tokyo, Japan) and an ultrasonic bath were used to prepare the nanofluids [58].

**Figure 8:**
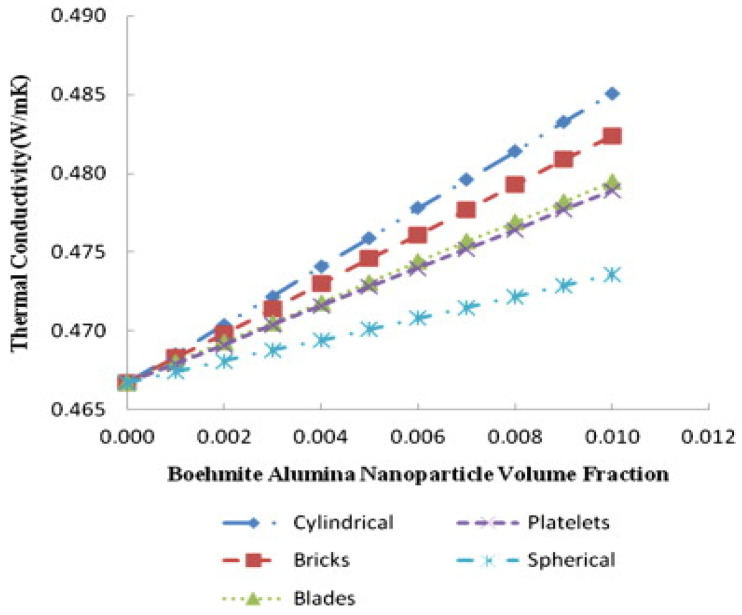
Effects of different particle shapes on the thermal conductivity of a nanofluid [59].

Elias et al. studied the effects of different forms of nanoparticles in nanofluids on heat exchanger performance. The results showed an increase in both system heat transfer and thermodynamic performance. Cylindrical nanoparticles showed better performance in terms of the thermal conductivity, heat transfer coefficient, and overall heat transfer coefficient (see Figure 8) [59].

Later, Shahsavar et al., in a numerical study, evaluated the thermal and hydraulic characteristics of a boehmite alumina nanofluid in a two-tube mini-channel heat exchanger considering various nanoparticle shapes. It was observed that morphology influences nanofluid thermal conductivity, where the cylindrical shape showed the best conductivity, and the platelet shape corresponded to the lowest thermal conductivity [60].

**Figure 9:**
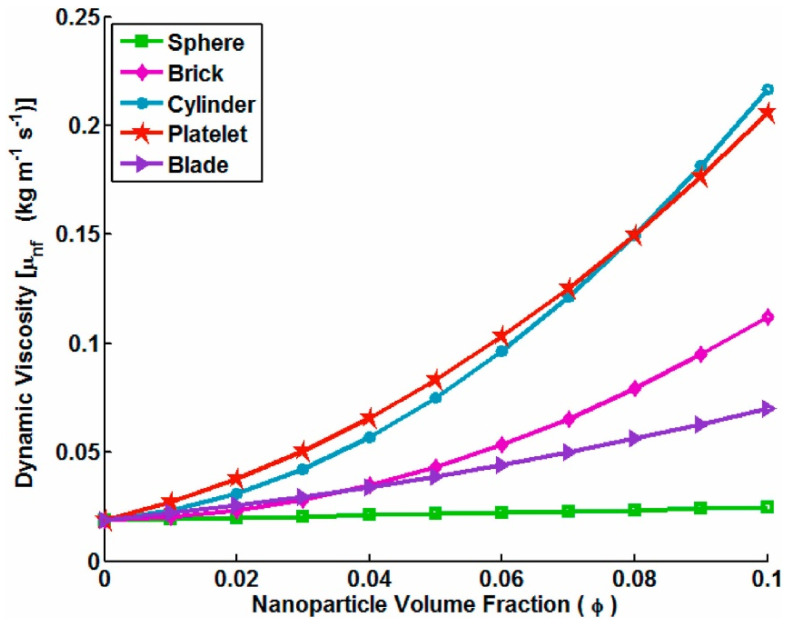
Shape effects on the dynamic viscosity of a nanofluid versus the nanoparticle volumetric concentration [61].

In a numerical study, Saranya et al., Dehaj et al., and Hajabdollahi et al. also found that the viscosity and thermal conductivity were influenced by nanoparticle shapes for different volume concentrations. In general, the nanofluid viscosity and thermal conductivity increase with increasing volumetric concentrations of particles, and the slope of the increase becomes higher with larger particle concentrations for different nanoparticle shapes. Nanofluid conductivities with cylindrical and brick-shaped NPs were higher at a fixed particle concentration compared to other forms of nanoparticles [61,62,63]. As can be seen, a higher viscosity was obtained using cylindrical and platelet nanoparticle shapes at a fixed particle concentration (Figure 9) [61].

In this section, the effects of nanoparticle shapes on the thermal conductivity and dynamic viscosity, among other thermophysical properties of nanofluids, were presented. Cylindrical nanoparticles always presented high performance for both properties mentioned above.

## 4. Effects of Nanofluid Characteristics on Heat Transfer Rate and Pressure Drop

In this section, the main characteristics that affect the heat transfer rate and pressure drop of a heat exchanger are presented. The equations below determine the heat transfer rate and pressure drop in a double-pipe heat exchanger. Nanofluid would be considered a working fluid. Each of the relevant nanofluid characteristics is analyzed, and its direct effects on the heat transfer and pressure drop are observed.

Subsequently, in the present review, the authors mathematically analyze these parameters with data extracted from [62] and determine the main nanofluid characteristics that influence both the heat transfer rate and pressure drop, thus further clarifying the context of this work. Heat exchanger dimensions are presented in Table 5. The nanofluid to be used is TiO_2_-water with 20 nm spherical nanoparticles. The nanofluid flows in the inner tube of the heat exchanger. The equations were solved using MATLAB.

### 4.1. Data Analysis

The average heat transfer rate in the heat exchanger is calculated from Equation (1) and presented with a ${\dot{Q}}_{HX}$ term [41]:

(1) $${\dot{Q}}_{HX}=UA\cdot \Delta T_{lm}=\frac{\Delta T_{lm}}{R_{HX}}$$

where *A* is the surface area of the inner tube, *U* is overall heat transfer coefficient of a double pipe, and ∆*T_lm_* is the logarithmic mean temperature difference for counter-flow conditions, and the thermal resistance is calculated according to Equation (2) [4,5]:

(2) RHX=1UA=1hnfAi+R″fiAi+ln(dodi)2πkLi+R″foAo+1hoAo

where *k* is the thermal conductivity of the tube material, and *A_o_* and *A_i_* are the inner and outer areas of the inner tube, respectively. *h_nf_* and *h_w_* represent the convective heat transfer coefficient of the nanofluid and water, respectively. ${R^{\prime \prime }}_{fi}\;\mathrm{and}\;{R^{\prime \prime }}_{fo}$ are fouling factors in the inner tube and the annular, respectively.

For this analysis, the nanofluid circulates in the heat exchanger inner tube. Consequently, it is enough to determine the nanofluid heat transfer coefficient because it affects the overall heat transfer rate of heat exchanger. The coefficient is calculated according to Equation (3):

(3) $$h_{\mathrm{nf}}=\frac{{\mathrm{Nu}}_{\mathrm{nf}}\cdot k_{\mathrm{nf}}}{d_{i}}$$

The dimensionless Nusselt number pertaining to nanofluids is evaluated, as follows, according to Equation (4) [4,64]:

(4) Nunf=3.66+0.065(diLi)RenfPrnf1+0.04[(diLi)RenfPrnf]2/3

The values of the Prandtl and Reynolds numbers for the nanofluid are determined by Equations (5) and (6). The Reynolds number is the factor that allows the determination of the fluid flow regime type. It depends on the nanofluid velocity, density, and viscosity. In this study, the flow regime is laminar.

(5) Renf=ρnf·unf·diμnf

(6) $${\mathrm{Pr}}_{\mathrm{nf}}=\frac{\mu_{\mathrm{nf}}\cdot {\mathrm{Cp}}_{\mathrm{nf}}}{k_{\mathrm{nf}}}$$

The pressure drop is directly related to the nanofluid velocity and the density and is calculated according to Equation (7) [41]:

(7) $$\Delta P_{\mathrm{nf}}{=f}_{\mathrm{nf}}\cdot \frac{\rho_{\mathrm{nf}}\cdot u_{\mathrm{nf}}^{2}}{2\cdot d_{i}}\cdot L_{i}$$

where Δ*P_nf_*, *ρ_nf_*, *u_nf_*, *f_nf_*, and *L_i_* are the nanofluid pressure drop, density, friction factor, velocity, and heat exchanger length, respectively. The friction factor is calculated by Equation (8) [5]:

(8) fnf=64Renf

The HTF pumping power (*P_pump_*) depends directly on the fluid pressure drop and the volumetric flow rate in the pipe and is required to calculate the amount of pump power to use. It is given by Equation (9) [41,65]:

(9) $$P_{pump}=\frac{\dot{m}\cdot \Delta P}{\rho }=\dot{V}\cdot \Delta P$$

Subramanian et al. experimentally analyzed the effects of the heat transfer and pressure drop of TiO_2_–water nanofluids flowing in a double-tube counter-flow heat exchanger. Their results showed that nanofluid heat transfer was greater than that of the base liquid (water) and increased with increasing Reynolds number and particle concentrations. The nanofluid pressure drop increased with an increment in volume concentration, being slightly higher than the base fluid [66]. Moreover, Saleh et al. noticed that the pressure drop is a function of the viscosity, volumetric flow rate, friction factor, and density as well as heat exchanger geometry. A higher pressure drop is noted for nanofluids due to their higher density [49].

In the next sections, thermophysical property effects are presented for the concentration and flow regime of a TiO_2_—water nanofluid. In addition, their influence on the heat transfer coefficient and pressure drop in the double-pipe heat exchanger is determined.

### 4.2. Effects of Nanofluid Thermal Conductivity on Heat Transfer Rate and Pressure Drop

Nanofluid thermal conductivity plays a key role in thermal efficiency for the double-pipe heat exchanger. Efficiency is influenced by several factors: the thermal conductivity, concentration, size, and morphology of nanoparticles. Equation (10) is commonly used to determine the nanofluid thermal conductivity of spherical nanoparticles [50].

(10) $$k_{\mathrm{nf}}{=k}_{\mathrm{bf}}\cdot \left[\frac{k_{\mathrm{np}}+2\cdot k_{\mathrm{bf}}-2\cdot \varphi \cdot \left(k_{\mathrm{bf}}-k_{\mathrm{np}}\right)}{k_{\mathrm{np}}+2\cdot k_{\mathrm{bf}}+\varphi \cdot \left(k_{\mathrm{bf}}-k_{\mathrm{np}}\right)}\right]$$

Considering a nanoparticle concentration variation from 0.0 to 0.8% in a base fluid with a constant flow of 0.4 L/min, according to Equation (10), the nanofluid thermal conductivity increases up to 1.98% greater than in the base fluid. It directly affects the heat transfer coefficient.

**Figure 10:**
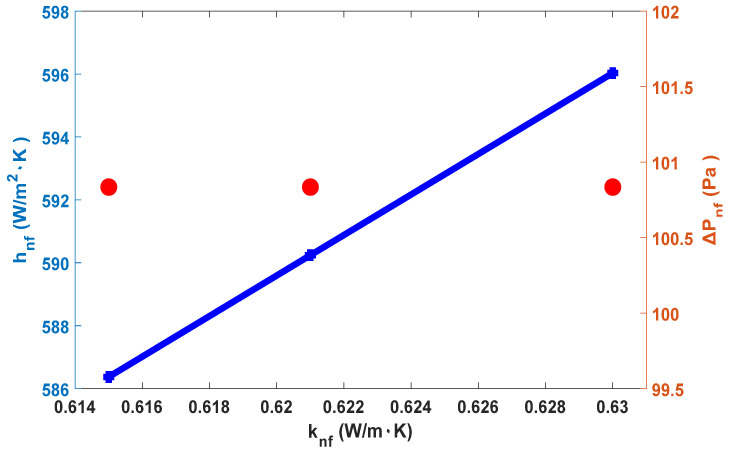
Heat transfer coefficient and pressure drop versus thermal conductivity enhancement (blue line represents heat transfer coefficient and red points represent pressure drop).

Depending on the nanofluid thermal conductivity enhancement and Equations (3) and (7), with a 0.4% concentration and a 0.5 L/min velocity, the heat transfer coefficient results in an improvement of 1.64%, and the pressure drop does not change (see Figure 10).

### 4.3. Effects of Viscosity of Nanofluids on Heat Transfer Rate and Pressure Drop

Viscosity is described as the resistance of one fluid layer against another layer of either fluid or solid [48]. Viscosity is considered another important factor for heat transfer applications. The pressure drop and pump power depend on it [28,65]. Viscosity is often determined experimentally. The nanofluid viscosity is inversely proportional to the temperature. It is determined according to Equation (11) [57]:

(11) $$\mu_{\mathrm{nf}}=\frac{\mu_{\mathrm{bf}}}{{\left(1-\varphi \right)}^{2.5}}$$

The nanofluid viscosity depends on some factors: the volume concentration, size, and shape of nanoparticles and the base fluid viscosity. Likewise, the shear rate and temperature affect the viscosity [28] as previously described in Section 4.1.

Considering a nanoparticle concentration variation from 0.0 to 0.8% in the base fluid according to Equation (11), the nanofluid viscosity increased up to 2.03% with respect to the base fluid.

**Figure 11:**
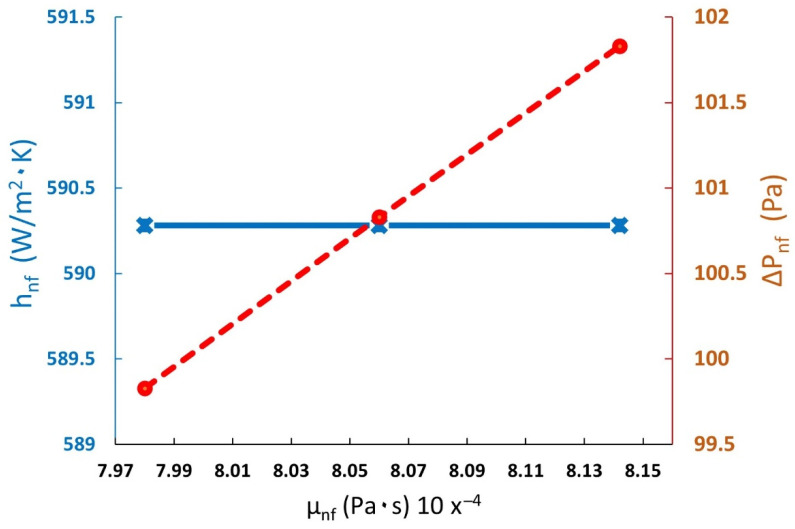
Heat transfer coefficient and pressure drop versus the nanofluid variation viscosity enhancement (blue solid line represents heat transfer coefficient and red dash-dotted line represents pressure drop).

Thus, depending on the nanofluid viscosity enhancement and Equations (3) and (7), with a 0.4% concentration and a 0.5 L/min velocity, the heat transfer coefficient stayed constant. The pressure drop was affected by an increase of up to 2.03% (see Figure 11).

### 4.4. Effects of Nanofluid Density on Heat Transfer Rate and Pressure Drop

In order to evaluate nanofluid heat transfer performance, density plays a key role. It directly affects the Reynolds number, friction factor, pressure drop, and Nusselt number [28].

Asokan et al. concluded that properties such as the pressure drop, friction factor, and Reynolds number depend on fluid density [47]. The nanofluid density is inversely proportional to the temperature. Very few researchers have studied this nanofluid property. The density is calculated using Equation (12). The nanofluid density is influenced by factors such as the size and concentration of nanoparticles and the temperature [28]. It also depends on the base fluid and the nanoparticle density. It influences both the heat transfer and the pressure drop.

(12) $$\rho_{\mathrm{nf}}=\left(1-\varphi \right)\cdot \rho_{\mathrm{fb}}+\varphi \cdot \rho_{\mathrm{np}}$$

In this data analysis, a nanoparticle concentration variation from 0.0 to 0.8% in the base fluid is considered. According to Equation (12), the nanofluid density results in an increase up to 2.61% greater than the base fluid.

Depending on the nanofluid density change percentage and Equations (3) and (7), with a 0.4% concentration and 0.5 L/min velocity, the heat transfer coefficient and pressure drop remain constant with their results unchanged.

### 4.5. Effects of Nanofluid Heat Capacity on Heat Transfer Rate and Pressure Drop

The specific fluid heat capacity determines the amount of heat absorbed to increase the temperature of 1 g of fluid by 1 °C. This is an important aspect of nanofluids used as refrigerants, as it affects heat transfer rate. The specific nanofluid heat capacity depends on the nanoparticle specific heat capacity [47]. The specific base fluid heat capacity is always greater than that of the nanofluid. It is determined according to Equation (13):

(13) $$C_{p,\mathrm{nf}}=\left[\frac{\varphi \cdot \left(\rho_{\mathrm{np}}\cdot C_{p,\mathrm{np}}\right)+\left(1-\varphi \right)\cdot \left(\rho_{\mathrm{fb}}\cdot C_{p,\mathrm{fb}}\right)}{\rho_{\mathrm{nf}}}\right]$$

The analysis shows that the nanofluid specific heat affects the heat transfer coefficient and does not affect the pressure drop. However, the influence is in a negative way because it results in a lower heat capacity than the base fluid. Thus, it is not significant for this process. Studies related to nanofluid specific heat capacity are not very advanced. However, fluid specific heat capacity is more important for thermal energy storage (TES) applications [47].

### 4.6. Effects of Nanoparticle Concentration on Heat Transfer Rate and Pressure Drop

The nanoparticle concentration is a basic and essential characteristic that influences nanofluid thermophysical properties. In addition, it affects the heat transfer rate and pressure drop. When the concentration increases, the nanofluid pressure drop and pumping power increase slightly, and the heat transfer coefficient can be enhanced.

Kavitha et al. experimentally studied the impacts of different concentrations of CuO nanoparticles in water as a base fluid on the heat transfer characteristics of a double-pipe heat exchanger with parallel flow. As a result, the average heat transfer coefficient achieved an increment of 2.12% with a volume concentration of CuO nanoparticles close to 0.004% [7]. Osman et al. experimentally investigated convection heat transfer in a transition flow regime of Al_2_O_3_–water nanofluids in a rectangular channel. The authors found that the heat transfer coefficient enhancement for the volume concentrations of 0.3%, 0.5%, and 1.0% nanofluids were 15%, 29%, and 54%, respectively. The pressure drop increase was significant in the transition flow regime, with values of 7.90%, 14%, and 61% for those nanofluid volume concentrations, respectively [46].

**Figure 12:**
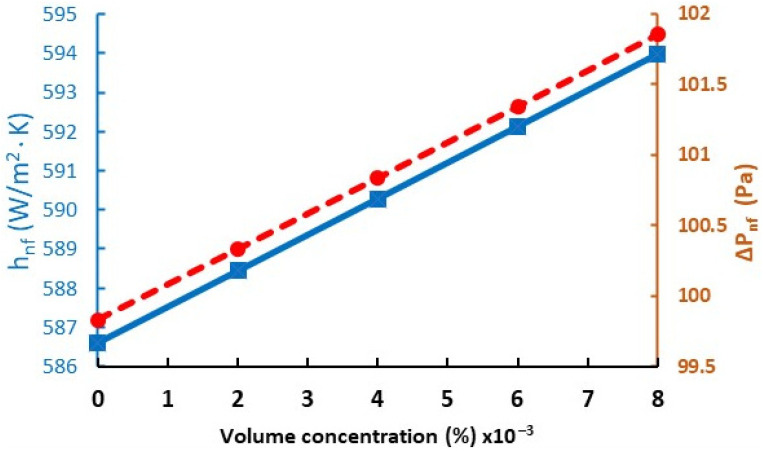
Heat transfer coefficient and pressure drop versus concentration variation (blue solid line represents heat transfer coefficient and red dash-dotted line represents pressure drop).

Considering nanoparticle volume concentration variations from 0.0 to 0.8% in the base fluid, with a constant volumetric flow of 0.5 L/min, the nanofluid thermophysical properties were affected by concentration, improving thermal conductivity by 1.98%, density by around 2.61%, and viscosity by 2.03%, while the specific heat decreased in by 2.80% compared to the base fluid. Hence, depending on nanofluid thermophysical properties percentage and Equations (3) and (7), it was observed that the heat transfer coefficient and pressure drop resulted in increases of up to 1.25% and 2.03%, respectively (see Figure 12 and Table 6).

According to the summary in Table 6, we can observe that with concentration (0.0–0.8%) variation, the heat transfer coefficient obtains an improvement of up to 1.25% accompanied by a 2.03% pressure drop increment. This increase in pressure drop is due to nanofluid viscosity, and the heat transfer is due to nanofluid thermal conductivity.

### 4.7. Effects of Nanofluid Flow Regime on Heat Transfer Coefficient and Pressure Drop

Flow regimes are classified into laminar (Re < ∼2100), transitional (∼2100 < Re < ∼4000), and turbulent (∼4000 < Re) based on fluid movements. This is determined according to Equation (5) by calculating the Reynolds number. The Reynolds number (Re) depends on three nanofluid characteristics, the density, dynamic viscosity, and velocity, defining the sort of flow regime. The Nusselt number is proportional to the Reynolds number, and the heat transfer coefficient increases directly with the Nusselt number (see Equation (4)). Additionally, the pressure drop also depends on the Reynolds number.

**Figure 13:**
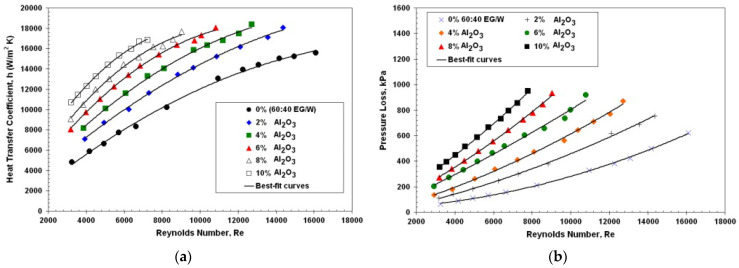
Variation in heat transfer rate (**a**) and pressure drop (**b**) with respect to the Reynolds number of nanofluid [39].

Bahmani et al. conducted a numerical study on forced convection in a double-tube heat exchanger using nanofluids (alumina–water) considering Reynolds numbers from 10,000 to 100,000. The results indicated that the Reynolds number caused an enhancement of the Nusselt number and the convection heat transfer coefficient. The maximum rate of the average Nusselt number and the thermal efficiency enhancement were 32.70% and 30%, respectively [67]. Later, Bahmani et al. carried out a study on forced convection in a double-tube heat exchanger using nanofluids with constant thermophysical properties, considering Reynolds numbers between 100 and 1500 and employing the finite volume method for solving the governing equations. They found that heat transfer rate could be enhanced by increasing the nanoparticle volume fraction and the Reynolds number [68]. Poongavanam et al. experimentally performed research on heat transfer and pressure drop using nanofluid multiwalled carbon nanotubes (MWCNT–Solar glycol) in a double-pipe heat exchanger. They observed that the average convection heat transfer coefficient of a nanofluid containing 0.6% MWCNT nanoparticles when the mass flow rate varied between 0.04 and 0.08 kg/s improved by ~21%, and average pressure drop increased up to 100% [41]. Similarly, Zhen et al. separately studied the use of different nanofluids (CuO–water, Al_2_O_3_–water, Fe_3_O_4_–water, ZnO–water, SiC–water, and SiO_2_–water) to improve a double-tube heat exchanger with Reynolds numbers in the range of 4500—14,500. Based on test results of heat transfer performance and flow resistance, the 1% CuO–water nanofluid showed a great advantage due to a relatively high heat transfer performance (44.3%) and a low friction factor (24.9~32.7%). The 0.5% SiO_2_–water nanofluids also presented a low friction factor (26.0~28.3%) with an enhancement of heat transfer of up to 32.5% [69,70] (here, the friction factor is proportional to the pressure drop). In another study, Subramanian et al. experimentally analyzed the heat transfer performance of a TiO_2_–water nanofluid in a double-pipe counter-flow heat exchanger for various flow regimes, laminar, transition, and turbulent cases, with Reynolds numbers between 1350 and 4650 and with a 0.5% volume concentration. It turned out that the heat transfer increased by 77%, and a 72% pressure drop increase was observed [66]. The heat transfer rate and pressure drop evolution along the inner tube are presented with respect to the Reynolds number for different concentrations of nanoparticles in Figure 13. The heat transfer and pressure drop increased with the nanofluid mass flow rate. Based on the flow, it can be seen that the heat transfer and pressure drop are affected by the Reynolds number, with a high percentage increment [39,45,66].

**Figure 14:**
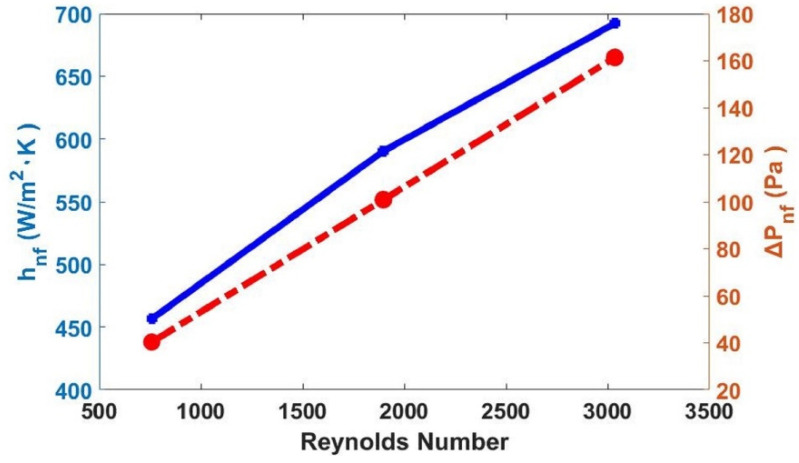
Heat transfer coefficient and pressure drop versus Reynolds number (blue solid line represents heat transfer coefficient and red dash-dotted line represents pressure drop).

In the mathematical study of this work, with volumetric flow rate variation (0.2–0.8 L/min) and a fixed concentration of nanoparticles (0.4%), the Reynolds number was augmented, as it was in laminar and transition regimes. In Figure 14, an increase in the Reynolds number was observed, which implied an increase of 17.20% in heat transfer and a 60% pressure drop. The pressure drop enhancement was much larger compared to the heat transfer rate increase, as presented in detail in the summary of Table 7. There, the pressure drop resulted in more than three times the percentage of the heat transfer coefficient.

In Table 8, a global summary of different influencing factors on nanofluid thermophysical properties and performance for a double-pipe heat exchanger is presented. All studies carried out in this review showed that increasing the nanoparticle volume fraction enhanced the thermal conductivity, dynamic viscosity, density, and heat transfer rate, with an increment in pressure drop. It was necessary to avoid a significant percentage of nanoparticle concentration to avoid heat exchanger leaks.

For the particle size, smaller nanoparticles enhanced the nanofluid thermal conductivity and viscosity. They provided improved heat exchanger heat transfer and high efficiency. They also allowed for easier nanofluid circulation, avoiding high pressure drop.

Cylindrical and brick shapes presented better conductivity. Cylindrical and platelet shapes of nanoparticles enhanced the viscosity and provided improvements in heat transfer. However, they resulted in a pressure drop increase. The flow regime depends directly on the flow velocity. Thus, when the velocity increases, the Reynolds number increases and enhances the heat transfer rate, with a pressure drop increase.

Finally, according to the data analyzed with the mathematic model, the nanoparticle volume concentration could improve the nanofluid density, viscosity, thermal conductivity, and heat transfer coefficient, while this is associated with an increase in pressure drop. Thus, it is clearly observed that nanofluid thermal conductivity is directly associated with the heat transfer coefficient, and viscosity is associated with pressure. The nanofluid density did not affect the heat transfer coefficient or the pressure drop; it did not cause any change in them.

## 5. Conclusions

This review is based on the analyses of various factors of nanoparticles on nanofluid properties, and the influences of nanofluid characteristics on the performance of the double-pipe heat exchanger are discussed.

The nanoparticle volume concentration affects each thermophysical property of the nanofluids, improves the heat transfer coefficient, and increases the pressure drop. The smallest nanoparticles improve the thermal conductivity and increase the dynamic viscosity of the nanofluids. The shapes of nanoparticles, such as cylindrical and platelets, allow an increase in the thermal conductivity of a nanofluid and improve the heat transfer coefficient of a heat exchanger. However, the cylindrical and platelet shapes cause a large pressure drop, while spherically shaped nanoparticles present a lower percentage pressure drop. The data analysis was realized for a double-pipe heat exchanger using the mathematical model. The 20 nm diameter spherical nanoparticles with water as the base fluid were considered. The TiO_2_-water nanofluid was used as a working fluid. The results show that the nanoparticle volume concentration displayed a significant influence on the nanofluid thermophysical properties. For a volume concentration of 0.8% nanoparticles, the thermal conductivity, viscosity, and density of the nanofluids increased by 1.98%, 2.03%, and 2.61%, respectively, compared to the base fluid. An impact on heat exchanger performance was achieved, with a constant volumetric flow rate of 0.5 L/min, obtaining an improvement in the heat transfer coefficient close to 1.25% and an increment in the pressure drop of 2.03%. Furthermore, with a constant volume concentration of 0.4% and a volumetric flow rate variation from 0.2 to 0.8 L/min, a high percentage in the pressure drop (60%) and an enhancement of 17.20% in the heat transfer coefficient were observed.

As a recommendation, it is advisable to select nanoparticles of high thermal conductivity, cylindrical shape, and small sizes. The concentration must adjust with the volumetric flow rate in order to avoid a large pressure drop. The percentage of pressure drop should not be greater than the percentage of improvement in the heat transfer rate.

## Figures and Tables

**Table 1:** Examples of methods used to improve the double-pipe heat exchanger.

Methods	Improvement Approach	Observations (by Percentage, %)	Ref.
Active	Using air bubble injection	The percentage improvement of the overall heat transfer coefficient can be from 10.30% to 149.50%.	[16]
	Using surface vibrations	Heat transfer coefficient enhancement of 9%	[17]
	Using magnetic field	Heat transfer enhancement up to 320% and a slight increase in pressure drop	[18]
	Using ultrasonic vibration	Heat transfer is enhanced by about 60%.	[19]
Passive	Porous media	Enhanced heat transfer of 44% with larger pressure drops	[20]
	Using fins	Heat transfer rate enhancement of around 90–98%. Pressure drops for finned tube also increased.	[21]
	Helical wires	Augments Nusselt number by up to 2.64 fold. Increases friction factor by about 2.74 fold.	[22]
	Twisted tape	Significant (15%) enhancement in heat transfer rate. Friction factor increased by 10%.	[23]
	Metal foam	Nusselt number enhanced by 57.21%.	[24]
	Nanofluids (MgO-ethylene glycol)	Heat transfer coefficient enhancement of 27% for wt.% = 0.3 and 35% pressure drop at wt.% = 0.3.	[25]

**Table 3:** Influences of the concentration of nanoparticles on nanofluid properties according to different studies.

Base Fluid	Nanoparticles	Particle Concentration (%)	Size (nm)	Temperature Range (°C)	Observation	Ref.
Ethylene glycol	MWCNT (multi-walled carbon nanotubes)	0.02–0.10	12–30	30–60	Thermal conductivity increased by 50% with a 0.06% concentration. Viscosity increased by 58% with a 0.1% concentration.	[51]
TH55 oil	Al_2_O_3_	0.10–1.00	40–50	20–90	Thermal conductivity was enhanced by 8.44% at 65 °C with a 1.0% concentration. Density increased 3.31% at 20 °C with a 1.00% concentration. Viscosity at 30 °C improved by 28%.	[52]
TH55 oil	GNP (graphene nanoplatelets)	0.01–0.10	--	20–90	Thermal conductivity was enhanced by 15.69% with a concentration of 0.10% at 65 °C. Density increased slightly compared to the base fluid. Viscosity at 30 °C improved by 32%.	[52]
60% Ethylene glycol/40% water	CuO	0.06	<50	<100	Thermal conductivity improved by 26%, density improved by 29%, and viscosity improved by 27%.	[47]
Water	Al_2_O_3_	1.00	20	55	Increased viscosity by 31% compared to the base fluid.	[45]
Water	MWCNT (multi-walled carbon nanotubes)	0.30	15	25–70	Thermal conductivity increased by 9.80–15.28%. Viscosity increased by 11.489.15%, and density increased as well.	[49]
Water	TiO_2_	0.25	8–18	30–60	Average thermal conductivity increased by 8.5%, 6.0%, and 5.0%. Average density increased by 7%, 11%, and 24%. Average viscosity increased by 4.7%, 5.1%, and 5.3%, respectively.	[40]
	ZnO		12–28			
	Ag		7–24			

**Table 4:** Influences of nanoparticle size on nanofluid properties according to different studies.

Base Fluid	Nanoparticles	Particle Concentration (%)	Size (nm)	Temperature Range °C	Observation about Nanofluids Properties	Ref.
Water	Al_2_O_3_	1.0%	20	10–50	Thermal conductivity of 20 nm was enhanced by 1.54% and 2.43% more than those of 50 nm and 100 nm, respectively. No significant variation in density.	[53]
			50			
			100			
Water	SiO_2_	--	15	25–65	Thermal conductivity was improved by 3.80, 4.90, and 7.80%, respectively, compared to the base fluid. The viscosities increased by 6.10, 8.30, and 9.20% compared with those of pure water.	[54]
			30			
			80			
Ionic liquid	Al_2_O_3_	1.0%	10	10–90	Thermal conductivity was enhanced by 9.73%, 6.53%, 6.41%, and 7.60% for 10 nm, 30 nm, 60 nm, and 90 nm nanoparticles, respectively. Density and viscosity did not have significant differences based on the sizes of nanoparticles.	[55]
			30			
			60			
			90			
Water	CuO	0.1%	20	20–70	Thermal conductivity of 25 nm (18%) was larger than the 50 nm size (15.60%) at 30 °C.	[30]
			50			
Engine oil	ZnO	7.5%	20	--	Viscosity increased with an increase in nanoparticle size by 3.80%, 4.61% 5.30%, 6.90%, and 9.23%, respectively.	[56]
			40			
			60			
			80			
			100			

**Table 5:** Double-pipe heat exchanger dimensions.

	Material	Inner Diameter (m)	Thickness (m)	Length (m)
Inner tube	Copper	0.007	0.00150	0.885
Annular	Galvanized iron	0.022	0.00275	0.885

**Table 6:** Summary of nanoparticle volume concentration variation effects on nanofluid thermophysical properties, heat transfer, and pressure drop of heat exchanger.

Concentration	0% (Base Fluid)	0.2%	0.4%	0.6%	0.8%	Observation
*k_nf_* (W/m. °C)	0.6150	0.6180	0.6211	0.6241	0.6272	Increased up to 1.98%
*µ_nf_* (10^−4^ Pa. s)	7.98 × 10^−4^	8.02 × 10^−4^	8.06 × 10^−4^	8.10 × 10^−4^	8.14 × 10^−4^	Increased up to 2.03%
*cp_nf_* (J/kg. °C)	4178	4148	4119	4090	4061	Decreased up to 2.80%
*ρ_nf_* (kg/m^3^)	996	1002	1009	1015	1022	Increased up to 2.61%
Flow rate (L/min)	0.5	0.5	0.5	0.5	0.5	One flow rate considered
*Re_nf_*	1891.10	1893.90	1896.70	1899.40	1901.90	Laminar regime
*h_nf_* (W/m^2^. °C)	586.66	588.43	590.28	592.12	594.04	Increased up to 1.25%
Δ*P_nf_* (Pa)	99.86	100.33	100.83	101.34	101.89	Increased slightly up to 2.03%

**Table 7:** Summary of nanofluid flow rate variation effects on flow regime, heat transfer coefficient, and pressure drop.

Nanofluid Flow Variation in Double-Pipe Heat Exchanger (TiO_2_–Water)				Observation
Concentration	0.4%	0.4%	0.4%	One percentage considered
Flow rate (L/min)	0.2	0.5	0.8	Variation
*Re_nf_*	758.68	1896.70	3034.70	Increased by 59.90%
*Nu_nf_*	5.14	6.65	7.80	Increased by 17.20%
*h_nf_* (W/m^2^. K)	456.60	590.28	692.10	Increasing up to 17.20%
Δ*P_nf_* (Pa)	40.33	100.83	161.33	Increasing up to 60%

**Table 8:** Overall summary of analysis of factors influencing nanofluid thermophysical properties and double-tube heat exchanger performance.

Affecting Factors	Analysis	Nanofluid Thermophysical Property Remarks	Double-Tube Heat Exchanger Performance Remarks
Concentration of nanoparticles	Increment in volume fraction	Improved thermal conductivity, density, and viscosity. Decreased specific heat.	Enhanced heat transfer rate. Increased pressure drop.
Size of nanoparticles	Various sizes analyzed	Smaller ones enhanced thermal conductivity. Viscosity slightly increased.	Smaller ones provided better heat transfer and high efficiency.
Shape of nanoparticles	Blades, platelets, cylindrical, cubic, spherical	Cylindrical and bricks (cubic) enhanced thermal conductivity. Cylindrical and platelets enhanced viscosity.	Cylindrical and platelets present better heat transfer but increase pressure drop. Spherical shape presented lower percentage of pressure drop.
Flow regime of nanofluids	Increasing the flow	Classification of the regime flow: Laminar, transition, and turbulent	Heat transfer rate enhanced when Reynolds number increased. Obtained a high pressure drop.
Thermophysical properties of nanofluids (density, viscosity, and thermal conductivity)	Experimental and mathematical model	--	Thermal conductivity enhanced heat transfer coefficient. Viscosity increased pressure drop.
